# Detection of Di- and Tri-Locus *kdr* Mutations in *Aedes aegypti* and *Aedes albopictus* from Texas, USA, and the Implications for Insecticide Resistance

**DOI:** 10.3390/insects16060551

**Published:** 2025-05-23

**Authors:** Bianca M. Wimmer, Cynthia Reinoso Webb, Steven M. Presley

**Affiliations:** Department of Environmental Toxicology, The Institute of Environmental and Human Health, Texas Tech University, Lubbock, TX 79409, USA; bianca.rendon@ttu.edu (B.M.W.); cynthia.reinoso@ttu.edu (C.R.W.)

**Keywords:** *Aedes aegypti*, *Aedes albopictus*, *kdr*, mosquito control, insecticide resistance, co-occurring mutations

## Abstract

During the last 20 years, there has been increasing concern about inefficient vector control efforts due to insecticide resistance. A common mechanism causing insecticide resistance is mutational changes in the voltage-gated sodium channel, deemed knockdown resistance (*kdr*), resulting from continued pyrethroid application. Although closely related, there have been documented *kdr* differences and frequencies between *Aedes aegypti* and *Aedes albopictus*. Individual *Ae. aegypti* and *Ae. albopictus* from five counties in Texas, USA were tested using four single nucleotide polymorphisms genotyping assays to assess the *kdr* (F1534C, V1016I, V410L, and S989P) differences between the two species. Each mutation was analyzed independently by calculating frequencies and analyzing the difference using a Wilcox Rank Sum test. Significant differences were observed between *Ae. aegypti* and *Ae. albopictus* when comparing F1534C and V410L (*p*-value < 0.0001). Knockdown resistant mutation V1016I was not different between the two species. Individuals from both species had di-locus mutations, and individuals from *Ae. aegypti* had tri-locus mutations detected in combinations that have been reported to influence insecticide resistance. Given our findings, one can speculate that populations of both species are resistant to pyrethroids, thus likely limiting the success of control methods.

## Simple Summary

Insecticide resistance caused by knockdown resistant (*kdr*) mutations is a growing concern for mosquito control. Different mutations and their rates of occurrence have been documented in *Aedes aegypti* and *Aedes albopictus*. Four mutations were assessed in mosquito populations from five Texas counties, USA. Differences between *Ae. aegypti* and *Ae. albopictus* were calculated and determined for *kdr* mutations F1534C and V410L, but there was no difference with the mutation V1016I. Furthermore, S989P was not detected in either species. Multiple combinations of di-locus and tri-locus mutations were detected in mosquitoes from all counties. These combinations can amplify insecticide resistance, limiting the effectiveness of vector control operations.

## 1. Introduction

Emerging and re-emerging infectious diseases are increasing globally, and many are vectored by arthropods [1]. It is estimated that 22% of all newly emerging diseases are caused by vector-borne zoonotic pathogens [2]. Furthermore, mosquito- and tick-borne diseases tripled during the 12-year period from 2004–2016 in the USA [3]. Within the USA, Texas has numerous mosquito species known to be vectors, as well as many arboviruses, due to the range of ecoregions, high immigration rates from bordering countries and states, and large metroplexes that create ideal habitats for anthropogenic species. Autochthonous transmission of West Nile virus (WNV), St. Louis encephalitis virus (SLEV), dengue virus (DENV), Eastern equine encephalitis virus (EEEV), chikungunya virus (CHIKV), Zika virus (ZIKV), and Western equine encephalitis virus (WEEV) are reported sporadically in Texas [4].

West Nile virus, SLEV, and EEEV pose the most zoonotic risk and are detected annually in human, animal, and/or mosquito samples [4]. To limit the spread of these viruses, control focuses on eliminating or reducing the vectors—*Culex tarsalis* (Coquillett), *Culex quinquefasciatus* (Say), and *Aedes vexans* (Meigen)—as well as other nuisance species. Chemical applications used to limit these vectors also expose nontargeted vectors, such as *Aedes aegypti* (Linnaeus) and *Aedes albopictus* (Skuse) that occur within the state [5]. Although this unintended exposure may help lower the risk of pathogens they vector (i.e., DENV, CHIKV, and ZIKV), it also increases the likelihood of mosquito populations developing insecticide resistance [5].

Approaches for controlling adult mosquito populations in the USA include the application of organophosphate and/or pyrethroid pesticides [6], but due to the limited number of chemical classes available and their rapid generation turnover times, insecticide resistance can develop [7]. Reports have documented pyrethroid resistance in *Ae. aegypti* and *Ae. albopictus* populations occurring in California, Florida, and New Mexico [8,9,10]. Pyrethroids inhibit the voltage-gated sodium channel (VGSC) from closing, causing continued stimulation [11]. Pyrethroid resistance results from target site modifications due to single nucleotide polymorphisms (SNPs) at amino acid positions within the VGSC and causes knockdown resistance (*kdr*) [12].

Within mosquitoes, SNPs at amino acid positions 410, 1016, 1534, and 1763 in the VGSC have largely been documented to cause phenotypic insecticide resistance [13]. These SNPs have been recorded as single, di-, and tri-locus mutations, with various combinations of mutations and substitutions influencing the level of resistance [12]. Another common mutation is at position 989 where the serine is substituted for a proline (Ser → Pro, S989P) [14]. Although common, this mutation has only been documented to influence phenotypic resistance in combination with other *kdr* mutations and has only been detected within *Ae. aegypti* and *Ae. albopictus* from Asia [15,16].

The first report of *kdr* mutations in *Ae. aegypti* from the USA was from California during 2016, where V1016I was identified in all individual mosquitoes tested [8]. Since that report, V1016I has been detected in Florida and Texas [9,17,18]. The *kdr* mutation F1534C has been detected in California, Florida, New Mexico, and Texas since 2018 [9,17,18]. V410L is another common mutation shown to cause phenotypic resistance, especially when co-occurring with other *kdr* mutations [19]. In California, V410L was reported as a single, di-, and tri-locus mutation [18], additional di- and tri-locus V410L mutations were identified in New Orleans, LA and Harris County, TX [20,21].

Discrepancies exist between the number of *kdr* mutations studied and identified in *Ae. aegypti* and *Ae. albopictus*. Marcombe and contributors first reported a novel mutation within the VGSC of *Ae. albopictus* at amino acid position 1534 with the amino acid substitution of the wildtype phenylalanine to the resistant-type leucine (Pre → Leu, F1534L) [22]. This mutation was not associated with pyrethroid resistance within the Florida population studied but was with DDT resistance [22]. More recently, a different substitution at site 1534, phenylalanine to serine (Pre → Ser, F1534S), was identified in *Ae. albopictus* from Florida and North Carolina [23,24]. As of late 2024, these three reports were the only known reports of *kdr* mutations within *Ae. albopictus* populations from the USA [16].

Various combinations of di- and tri-locus *kdr* mutations have been reported, with combinations involving F1534C and V1016G/I being the most common [13]. In the USA, F1534C and V1016I have been reported primarily in Florida with a few reports from Harris County, TX [9,17]. It is important to understand which mutations are occurring and if any are co-occurring given that certain mutation combinations intensify phenotypic pyrethroid resistance [13].

The present study was conducted to determine if four *kdr* mutations—three that have been documented in the USA (V410L, V1016I, and F1534C) and one that has not (S989P)—occur in Texas populations of *Ae. aegypti* and *Ae. albopictus*, while also determining if there are differences in *kdr* frequencies among species.

## 2. Materials and Methods

### 2.1. Mosquito Collection and Rearing

**Figure 1:**
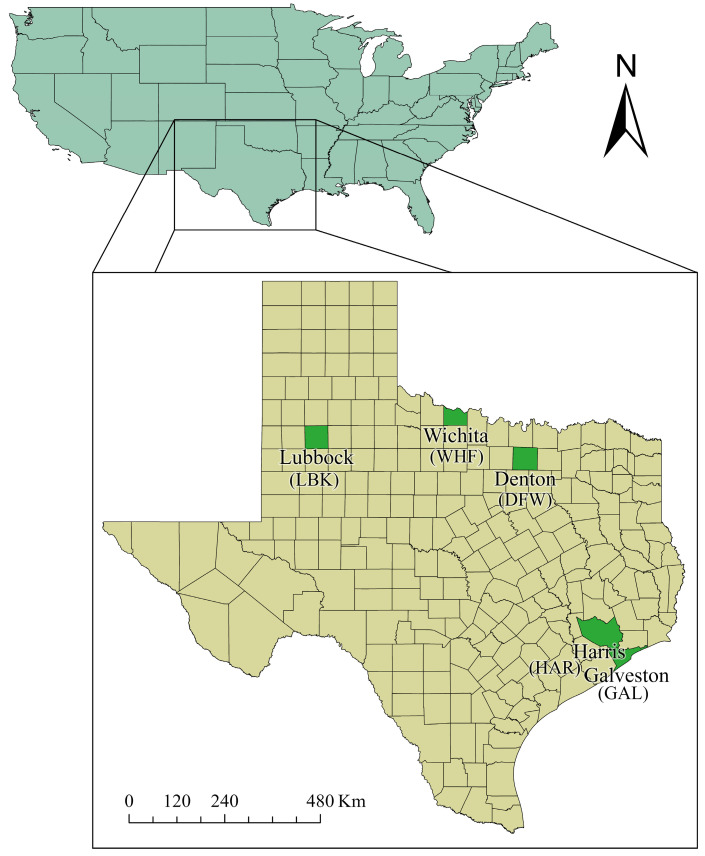
Participating counties with *Ae. aegypti* and *Ae. albopictus* collection sites.

*Aedes aegypti* and *Ae. albopictus* were collected simultaneously during three years (2017–2019) at various locations within five Texas counties (Figure 1). Counties included Denton County (DFW), Galveston County (GAL), Harris County (HAR), Lubbock County (LBK), and Wichita County (WHF). Each county voluntarily deployed ovitraps for four-days, with six deployments per mosquito season (May–September 2017, 2018, and 2019). Six ovitraps were deployed at locations of no less than 1 km separation from each other within each county. Ovitrap locations varied with each deployment. Ovitraps were fitted with germination paper and filled with water following methods reported by Peper and colleagues [25]. Germination papers were collected following each four-day egg collection period and stored in darkness until shipped to the Texas Tech University Vector-borne Zoonosis Laboratory for rearing and testing.

Germination papers received at the laboratory were immediately examined for eggs. Rearing was initiated when approximately 200 eggs combined across ovitraps had been received from an individual county each year. Mosquito eggs were carefully removed from the germination paper into a rearing tray (33 cm × 25 cm × 7 cm) filled with approximately 750 mL of DI water. The rearing tray was then placed within an insectary maintained at 70% (±5%) RH and 26 °C (±1 °C) with a 12:12 light:dark cycle [26]. All eggs were started within a six-week window of initial collection to limit desiccation and ensure adequate hatching rates [27]. Larvae were maintained on a TetraMin fish diet (Tetra-Fish, Blacksburg, VA, USA) slurry daily; the amount fed was dependent on the size and number of larvae within the rearing tray. Fourth instar larvae and pupae were transferred to rearing cages (Megaview Science Co., Ltd., Taichung, Taiwan; 30 cm × 30 cm × 30 cm) and provided 10% sucrose as a food source.

### 2.2. DNA Extraction

Adult three-to-five-day old F_1_ mosquitoes were sexed, speciated, and stored at −20 °C for up to one year, then transferred to −80 °C until extractions and PCR could be performed. Thirty adult female and male aedine mosquitoes from each county and year were randomly selected and used for *kdr* testing. The number of individuals tested from each species was dependent on the number of mosquitoes available. For example, if only a limited number of *Ae. aegypti* were available from a county during a given year, more *Ae. albopictus* were tested, and vice versa. In total, 389 mosquitoes were extracted and tested for *kdr* mutations (151 *Ae. aegypti* and 238 *Ae. albopictus*). Individual mosquito DNA was extracted using Qiagen’s DNeasy^®^ Blood and Tissue Kit (Qiagen, Hilden, Germany) [10]. A more specific insect DNA extraction protocol was followed to ensure adequate amounts of total DNA were obtained [28]. DNA was quantified using BioTek Synergy™ Neo2 (Agilent Technologies, Inc., Santa Clara, CA, USA) and samples were diluted to a standard 20 ng/µL using molecular grade H_2_O [29]. Only samples with high 260/280 ratios (~1.8) were utilized for genotyping.

### 2.3. Knockdown Resistance Genotyping

Following DNA extractions and standardization, *kdr* mutations in individual mosquitoes were assessed using four independent TaqMan^®^ SNP Genotyping Custom assays (Life Technologies Corporation, Carlsbad, CA, USA) [30]. The assays amplify and detect predetermined SNPs using TaqMan 5′-nuclease chemistry. Results are determined by FAM and/or VIC dyes fluorescing when a specific allele is amplified, signaling the call. These assays were originally developed for *Ae. aegypti* but were utilized for *Ae. albopictus* as well, with the idea of creating a high throughput method to investigate *kdr* mutations utilizing tools available for local vector control districts. Three independent assays were developed using mosquitoes from Brazil and tested for the *kdr* mutations V410L, V1016I, and F1534C [29]. One additional assay was developed for *kdr* mutation S989P in Pakistan [31]. Utilizing the S989P assay provided an opportunity to assess a novel mutation not regularly identified within the Western Hemisphere.

TaqMan^®^ SNP Genotyping assays operate via a real-time PCR approach, with pre- and post-PCR allelic discrimination plate reads. Reactions consisted of 12.5 µL of 2× TaqMan^®^ master mix (Life Technologies Corporation, Carlsbad, CA, USA), 1.25 µL of 20× assay stock, and 11.25 µL of diluted sample as detailed within the assay’s protocol. All reactions were completed using an ABI 7500 Dx (Applied Biosystems™, Foster City, CA, USA) following an enzyme activation phase of 95 °C for 10 min, an additional 95 °C for 15 s denaturation, plus 60 °C for a minute for the final annealing and extension phases over a combined 40 cycles. Post-PCR reads were saved and analyzed using the TaqMan^®^ GenoTyper Software V1.6.0. Possible calls included homozygote wild type (non-*kdr* type), heterozygote, homozygote (*kdr* type), undetermined (results did not analyze as either the wild type, heterozygote, or *kdr* type), and no amplification. Undetermined and no amplification calls were considered unsuccessful failures.

### 2.4. Analysis

Mutation frequencies were calculated separately for each *kdr* mutation. The Wald interval was utilized to calculate a 95% confidence interval following Agresti and Coull [32] and Alvarez and colleges [33]. Wilcoxon Rank-Sum tests were used to compare species differences in overall *kdr* frequencies for each mutation, and the significance was determined as a *p*-value < 0.05 (File S1). Figure 1 was created using ArcGIS Pro (v 3.2.2). Data analyses and remaining figures were completed in R Studio (v. 2024.04.1 Build 748).

## 3. Results

Insecticide resistance was not assessed during this project, yet unpublished data is available for resistance statuses of these populations. Permethrin resistance was recorded in DFW, GAL, HAR, LBK, and WHF. Deltamethrin resistance was recorded in WHF as well.

### 3.1. Species Differences

**Figure 2:**
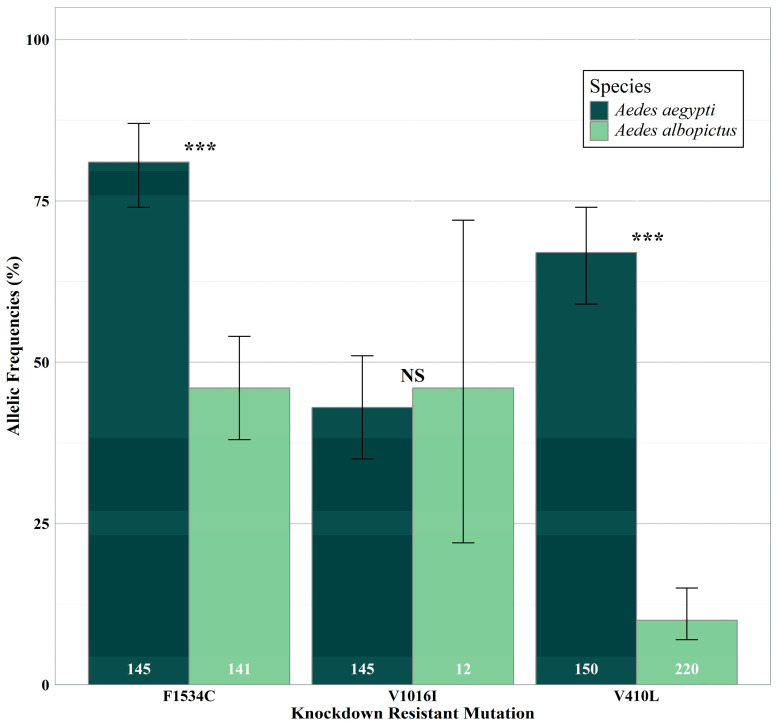
Differences in *kdr* mutation detection frequency between *Ae. aegypti* and *Ae. albopictus*. Labels indicate significant differences between the species for the given mutation. 95% confidence intervals are depicted by each bar. *** = *p*-value < 0.0001; NS = Not Significant.

Overall, *kdr* mutations were detected at higher frequencies in *Ae. aegypti* when compared to *Ae. albopictus* (Figure 2), partially due to a high number of failures within *Ae. albopictus* samples. Understandably, given the lower frequency of *kdr* mutations, *Ae. albopictus* had a higher number of completely susceptible (non-heterozygous) individuals (81% of *Ae. albopictus* compared to only 9% of *Ae. aegypti*). The *kdr* mutation S989P was not detected in mosquitoes from any county. This *kdr* mutation was not included in additional analyses due to the lack of result differences. There was a significant difference in *kdr* F1534C detection (*p*-value < 0.0001), in which it occurred in 81% of *Ae. aegypti* individuals compared to 46% of *Ae. albopictus* individuals. When successful, V1016I was detected similarly across both species (43% within *Ae. aegypti* and 46% within *Ae. albopictus* populations; *p*-value = 0.209). The *kdr* mutation V410L was detected in 67% of *Ae. aegypti* compared to 10% of *Ae. albopictus* (*p*-value < 0.0001).

Additionally, the homozygous di-loci and heterozygous combination (CC+VI+LL) was detected in 28% of *Ae. aegypti*. This combination was also seen in *Ae. albopictus* but at a much lower frequency (2% of individuals). The homozygous tri-loci CC+II+LL combination was detected in 10% of *Ae. aegypti*, while it was not detected in *Ae. albopictus*.

### 3.2. Aedes aegypti

**Figure 3:**
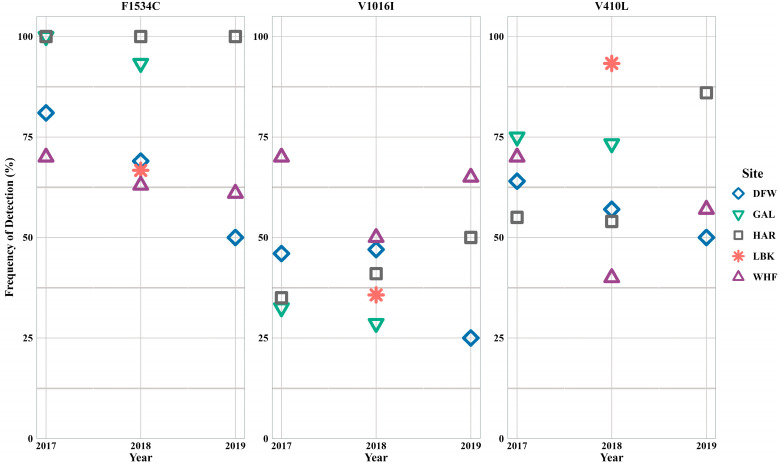
*Aedes aegypti kdr* frequencies for amino acid positions 1534, 1016, and 410.

The level of fully susceptible individuals, the frequency of *kdr* mutations, and the frequency of di-and tri-locus mutations in *Ae. aegypti* differed in each county (Table 1). Additionally, results varied between the years among the counties (Figure 3). Knockdown resistant mutation F1534C was observed at the highest frequency compared to the other mutations. All populations had more than 50% F1534C frequency.

In DFW mosquitoes, F1534C and V410L steadily decreased from 2017 to 2019, although only two individuals from 2019 were available and tested. The *kdr* mutation V1016I stayed consistent during 2017 and 2018 but decreased in 2019. The homozygous di-loci and heterozygous combination CC+VI+LL was detected during 2017 and 2018, although it was detected at a lower frequency in 2018 (38% in 2017 and 13% in 2018). An individual with the homozygous tri-loci mutation CC+VI+LL was detected only during 2017.

Knockdown resistance mutations did not differ greatly between 2017 and 2018 in GAL populations. All three mutations were detected at a frequency greater than 29%. Every tested individual during 2017 had the F1534C mutation. The homozygous di-loci and heterozygous combination CC+VI+LL was detected in both years at 50% and 53%, respectively.

Populations from HAR had 100% detection of F1534C during the three-year study. The frequencies of V1016I and V410L increased in HAR mosquitoes each year. The *kdr* mutation V1016I increased gradually from 35% during 2017 to 50% during 2019. Unlike the gradual increase of V1016I, V410L did not increase until 2019, increasing from 54% to 86%. The homozygous di-loci and heterozygous combination CC+VI+LL was detected each year, occurring at its highest (71%) during 2019. Like DFW samples, CC+VI+LL detection decreased during 2018 (2017: 40%; 2018: 33%), before increasing in the final year.

Only one year of data from LBK for *Ae. aegypti* was available for this assessment. The *kdr* mutation V410L was detected at its highest frequency in LBK (93%). V1016I was detected in 36% of the samples and F1534C was detected in 67% of the samples. Five samples (33%) had the homozygous tri-loci *kdr* mutation CC+II+LL.

Knockdown resistance mutations fluctuated in WHF samples. F1534C decreased from 2017 to 2019 (70%, 63%, and 61%, respectively), while V1016I decreased from 70% to 50% between 2017 and 2018 before increasing to 65% during 2019. The V410L mutation had a similar pattern as V1016I, decreasing from 70% during 2017 to 40% during 2018 and increasing to 57% during 2019. The homozygous tri-loci *kdr* mutation was detected during the three years following the same pattern as V1016I and V410L. Unlike the other counties, WHF was the only location to have CC+II+LL detected each year of the study. Interestingly, even though *kdr* mutations decreased from 2017 to 2018, the number of fully susceptible individuals also decreased, due to more individuals with heterozygous combinations.

### 3.3. Aedes albopictus

As previously stated, *Ae. albopictus* results were unlike the *Ae. aegypti* results (Figure 2). The frequency of fully susceptible individuals was higher in *Ae. albopictus* when compared to *Ae. aegypti*. The number of fully susceptible individuals decreased annually in DFW (2017: 100%; 2018: 38%; 2019: 26%). One homozygous di-loci and heterozygous combination was identified (CC+VI+LL) in HAR during the last year of the study (Table 2). This individual mosquito was the only sample from HAR that was not fully susceptible and had a *kdr* mutation. During 2017 and 2018, LBK had 93% and 87% fully susceptible individuals. The only variation within LBK *Ae. albopictus* populations were heterogeneous VI combinations during 2017 and 2018. Fully susceptible individual frequencies slightly fluctuated annually in WHF, occurring at high frequencies each year (2017: 96%; 2018: 100%; 2019: 93%).

Not only did populations have higher percentages of fully susceptible individual *Ae. albopictus*, but there were fewer samples with homozygous *kdr* mutations. When examining V410L, DFW had VL identified during 2018 and 2019, leading to 31% and 38% *kdr* frequency. In GAL, two individuals (14% of samples during 2018) had a CC+VV+VL combination and three individuals (21%) had a CC+VI+LL combination. The heterozygous combination VI was identified during 2017 and 2018 in LBK, resulting in 50% V1016I detection. During 2017, WHF had a singular homozygous *kdr* mutation (II, V1016I) identified. Additionally, the FC+VI+VL combination was observed in one individual from 2019 WHF specimens. No other combinations were observed in *Ae. albopictus*.

**Figure 4:**
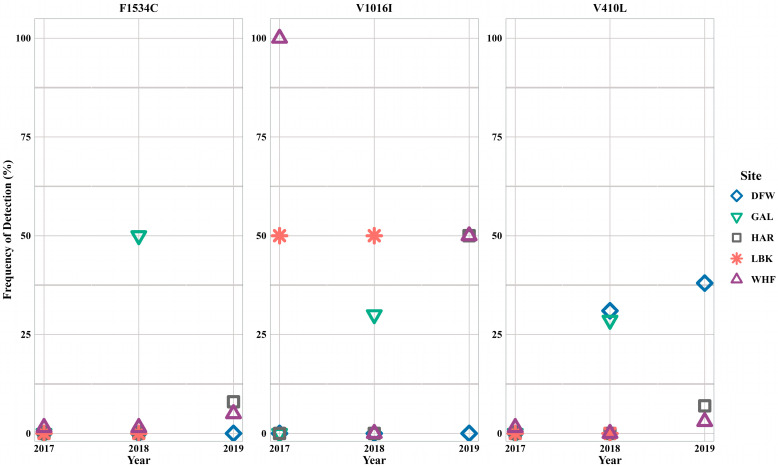
*Aedes albopictus kdr* frequencies for amino acid positions 1534, 1016, and 410.

Species-level yearly and county differences in *kdr* mutation detection were unattainable given the high number of fully susceptible individuals. The largest change in *kdr* mutation detection was in WHF across all three years for the V1016I mutation (Figure 4). Knockdown resistant mutation V1016I was detected at 100% during 2017, decreasing to 0% during 2018, and increasing to 50% during 2019. Detection frequencies increased from 2017 to 2018 for all three mutations in GAL.

## 4. Discussion

Four *kdr* mutations were assessed to determine if there were any *kdr* mutations and species differences between *Ae. aegypti* and *Ae. albopictus* populations from Texas. Three mutations (F1534C, V1016I, and V410L) were detected within these two species, while the fourth mutation (S989P) was not detected. Primarily detected in Asia, S989P was unlikely to be detected within local mosquitoes [15]. One of the underlying goals of this study was to detect emerging *kdr* mutations using available tools. Ovitraps were primarily placed in urban residential areas and parks where insecticide use is limited to household applications and vector control operations. Lack of immigration of foreign *Ae. aegypti* and *Ae. albopictus* and low selection pressure has limited this mutation from occurring at the time of this study. Even though the S989P mutation was not detected, the assay successfully produced homozygous and heterozygous results within both species. As such, this assay is an additional *kdr* detection tool that can be used by vector control districts that have real-time PCR capabilities.

Species-level differences in *kdr* frequencies were observed with the F1534C and V410L mutations. It has been well documented that *Ae. albopictus* has fewer known *kdr* mutations than *Ae. aegypti* [15]. Previous studies have indicated that *kdr* mutations are far less common in *Ae. albopictus*, potentially due to differences in insecticide exposure, selection pressure, and adaptation rates [15,34]. Previous reports have shown that there are more variations of *kdr* mutations in *Ae. albopictus* at single loci compared to *Ae. aegypti* [35]. For example, within *Ae. aegypti* the common substitution at position 1534 is Phe → Cys, yet within *Ae. albopictus* Phe → Cys, Phe → Leu, and Phe → Ser have been documented [16,24,35]. Additionally, these substitutions are not geographically isolated as seen within *Ae. Aegypti*, exhibited by V1016I in the New World and V1016G in the Old World [15]. The three substitutions at amino acid position 1534 (F1534C/L/S) detected in *Ae. albopictus* occur in the USA [35]. All three substitutions that have been reported in the USA have been shown to cause resistance to both Type I and Type II pyrethroids [16]. The substitution F1534C has been documented to cause resistance to permethrin and cyfluthrin (a Type II pyrethroid more commonly used in households) [16].

Successful results varied between the two species. *Aedes aegypti* produced successful results throughout the study, while *Ae. albopictus* did not, independent of the assay being performed. It is difficult to explain the high failure rate with *Ae. albopictus*. The specimens used in the study were collected, reared, stored, extracted, and tested concurrently by the same individual throughout the project. Previous genetic analysis at amino acid positions 989 and 1016 showed that these two species are identical [15], so using assays originally developed for *Ae. aegypti* does not explain the high failure rate within *Ae. albopictus*. As mentioned, there is a greater breadth of substitutions occurring at one amino acid position in *Ae. albopictus* [35]. Potentially, the reason for the high failure rate could be attributed to a focus on an incorrect substitution. The only method to rectify this pitfall is to sequence individual *Ae. albopictus* from across the state to understand which substitutions are truly occurring. Sequencing was beyond the scope of this study but should be incorporated into any future studies.

During this study, no differences were detected with the V1016I mutation, differing from the other two mutations. It must be noted that all analyses included only successful results. All results that could not be determined or failed were omitted from analyses. With a lower number of successful results with *Ae. albopictus* individuals, *kdr* frequency was calculated at a higher rate. Both species had more than 141 successful results for F1534C and V410L assays; however, only 12 *Ae. albopictus* results were successful with the V1016I assay. The lower number of successful results inflates the *kdr* frequency, resulting in findings that do not depict the actual occurrence. It is important to note that these failures included both no amplification and undetermined calls, further limiting the findings of this assay. Undetermined results can be explained by technical limitations of the primers, i.e., looking for the wrong substitutions as mentioned above. On the other hand, no amplification is more difficult to explain. All four *kdr* assays were used for every individual mosquito. If extractions were unsuccessful and DNA quality was poor, the number of failures due to no amplification would be the same for the other assays, which did not occur. Additionally, *Ae. albopictus* individuals that failed were retested to ensure the accuracy of the results. Results did not differ from the original, thus failure due to PCR plating errors is not a viable reason as well.

These findings can be useful for vector control districts because many di- and tri-locus mutations were detected. It is well documented that *kdr* mutations that occur together are common and may enhance insecticide resistance [36]. For example, F1534C as a single *kdr* mutation occurs across most of the globe and causes low levels of permethrin resistance, but in combination with V1016G resistance levels intensify [37]. Using *Xenopus* oocyte systems, co-occurring F1534C and V1016I will cause phenotypic resistance to Type I and Type II pyrethroids in *Ae. aegypti* [38]. To our knowledge, no data is available on the effect of di-locus mutations in *Ae. albopictus*. We hypothesize that if both mutations cause insecticide resistance as a single locus mutation, then when in combination changes to insecticide resistance levels would be similar to that of *Ae. aegypti*.

Tri-locus *kdr* mutations of various combinations are becoming more common. Individual *Ae. aegypti* had homozygous tri-locus mutations of F1534C+V1016I+V410L. This specific combination was first documented during 2018 from populations in Colombia, Mexico, and more recently in Harris County, TX [13,21]. In the original Mexico specimens, this combination strongly conferred knockdown resistance to Type I and Type II pyrethroids compared to other genotype combinations [39]. *Aedes aegypti* with this tri-locus mutation from Harris County, TX had increased survivorship following Permanone application, suggesting an influence on phenotypic insecticide resistance [21].

Given that mosquito populations from across the state tested in this study had di- or tri-locus mutations, effectiveness of vector control operations may be limited. Adulticidal pesticides within the USA have primarily relied on the use of two chemical classes, organophosphates and pyrethroids [5]. Kondapaneni and colleagues reported that the top four insecticide products utilized across the state of Florida were Naled, permethrin, permethrin + piperonyl butoxide, and malathion [40]. Through personal communication with the leadership from the various submitting vector control districts participating in this study, permethrin, malathion, and deltamethrin were the reported as the top three insecticides used. Exact rates of applications and/or application strategies were not provided. Excluding malathion (an organophosphate), the two pyrethroids are unlikely to be effective against these mosquito populations. Permethrin, a Type I pyrethroid, and deltamethrin, a Type II pyrethroid, would both be ineffective for controlling *Ae. aegypti* with the F1534C+V1016I+V410L combination [39]. Effective control will likely rely solely on malathion, which can result in organophosphate resistance that may already be present in the population. It must be noted that these results are limited due to the lack of phenotypic resistance data and rely solely on linking previous resistance studies with results reported from this study. Future studies need to include phenotypic bioassays and sequencing of the VGSC domains to get a complete understanding of *kdr* resistance across Texas.

## 5. Conclusions

Knockdown resistant mutations have been shown to differ between species. Fewer mutations were seen within *Ae. albopictus* compared to *Ae. aegypti*. Additionally, differences in *kdr* combinations were observed between the species. These differences were to be expected, as exposure pressure and genetic variability differ between the species.

Multiple di- and tri-locus mutations were observed, suggesting a higher probability of phenotypic pyrethroid resistance. Ultimately this data implies resistance status of these species without the use of resistance testing, and can be used by local vector control districts to enhance their control decisions, as multiple di- and tri-locus mutations were identified. Overall, this testing is a valuable tool for local vector control districts with RT-PCR capabilities.

## Figures and Tables

**Table 1:** Assay results for *Ae. aegypti* populations, divided by county and collection year. The number of individuals per result equals n and percentages are by year within each county.

Location	DFW			GAL		HAR			LBK	WHF			Total
**Year**	2017	2018	2019	2017	2018	2017	2018	2019	2018	2017	2018	2019	
Genotypes *	n (%)	n (%)	n (%)	n (%)	n (%)	n (%)	n (%)	n (%)	n (%)	n (%)	n (%)	n (%)	n (%)
**CC+II+LL**	1 (8%)	-	-	-	-	-	-	-	5 (33%)	3 (60%)	1 (7%)	5 (33%)	15 (10%)
CC+II+vv **	-	-	-	-	-	-	-	-	-	-	2 (13%)	-	2 (1%)
**CC+VI+LL**	5 (38%)	2 (13%)	-	10 (50%)	8 (53%)	4 (40%)	4 (33%)	10 (71%)	-	-	-	-	43 (28%)
**CC+VI**	2 (15%)	-	-	-	-	-	-	-	-	-	-	-	2 (1%)
**CC+VI+vL**	1 (8%)	3 (20%)	-	3 (15%)	-	3 (30%)	5 (42%)	4 (29%)	-	-	3 (20%)	-	22 (15%)
**CC+VI+vv**	-	-	-	-	-	-	-	-	-	-	1 (7%)	-	1 (<1%)
**CC+VV+vL**	-	-	-	7 (35%)	6 (40%)	-	-	-	1 (7%)	-	-	-	14 (9%)
**CC+VV+vv**	-	-	-	-	-	1 (10%)	-	-	-	-	-	-	1 (<1%)
**FC+VI+vL**	-	9 (60%)	1 (50%)	-	-	-	-	-	-	1 (20%)	5 (33%)	7 (47%)	23 (15%)
**FC+VI+vv**	1 (8%)	-	-	-	-	-	-	-	-	-	-	-	1 (<1%)
**FC+VV+LL**	-	-	-	-	-	-	-	-	7 (47%)	-	-	-	7 (5%)
**FC+VV**	-	1 (7%)	-	-	-	-	-	-	-	-	-	-	1 (<1%)
**FC+VV+vL**	1 (8%)	-	1 (50%)	-	-	-	-	-	1 (7%)	-	-	-	3 (2%)
**FC+VV+vv**	1 (8%)	-	-	-	-	-	-	-	-	-	-	-	1 (<1%)
**FF+LL**	-	-	-	-	-	-	-	-	1 (7%)	-	-	-	1 (<1%)
**FF+VV+LL**	-	-	-	-	-	-	-	-	-	-	1 (7%)	-	1 (<1%)
**FF+VV+vv**	-	-	-	-	-	-	-	-	-	1 (20%)	2 (13%)	1 (7%)	4 (3%)
**FF+vv**	1 (8%)	-	-	-	1 (7%)	-	-	-	-	-	-	1 (7%)	3 (2%)
**VV+vv**	-	-	-	-	-	2 (20%)	2 (17%)	-	-	-	-	-	4 (3%)
**vv**	-	-	-	-	-	-	1 (8%)	-	-	-	-	1 (7%)	2 (1%)
**Total**	13	15	2	20	15	10	12	14	15	5	15	15	151

* Genotypes represent potential combinations not including unsuccessful results. In cases with less than three combinations, the missing mutations were unsuccessful. ** Lowercase vv used to distinguish between V1016 and V410, with V410 being lower case.

**Table 2:** Assay results for *Ae. albopictus* populations, divided by county and year of collection. The number of individuals per result equals n, and percentage is by year within each county.

Location	DFW			GAL		HAR			LBK		WHF			Total
**Year**	2017	2018	2019	2017	2018	2017	2018	2019	2017	2018	2017	2018	2019	
Genotypes *	n (%)	n (%)	n (%)	n (%)	n (%)	n (%)	n (%)	n (%)	n (%)	n (%)	n (%)	n (%)	n (%)	n (%)
**CC+VI+LL**	-	-	-	-	3 (21%)	-	-	1 (7%)	-	-	-	-	-	4 (2%)
**CC+VV+vL**	-	-	-	-	2 (14%)	-	-	-	-	-	-	-	-	2 (1%)
**FC+VI+vL**	-	-	-	-	-	-	-	-	-	-	-	-	1 (7%)	1 (<1%)
FF+VI+vv **	-	-	-	-	-	-	-	-	2 (7%)	2 (13%)	-	-	-	4 (2%)
**FF+vv**	12 (75%)	1 (8%)	6 (22%)	5 (56%)	5 (36%)	13 (65%)	9 (50%)	11 (73%)	18 (60%)	13 (87%)	18 (75%)	9 (64%)	9 (60%)	129 (56%)
**FF+vL**	-	1 (8%)	10 (37%)	-	-	-	-	-	-	-	-	-	-	11 (5%)
**II+vv**	-	-	-	-	-	-	-	-	-	-	1 (4%)	-	-	1 (<1%)
**FF**	-	-	1 (4%)	-	-	-	-	-	-	-	-	1 (7%)	-	2 (1%)
**vv**	4 (25%)	4 (31%)	-	4 (44%)	4 (29%)	7 (35%)	9 (50%)	3 (20%)	10 (33%)	-	5 (21%)	4 (29%)	5 (33%)	59 (26%)
**vL**	-	7 (54%)	10 (37%)	-	-	-	-	-	-	-	-	-	-	17 (7%)
**Total**	16	13	27	9	14	20	18	15	30	15	24	14	15	230 ***

* Genotypes represent potential combinations, not including unsuccessful results. In cases with less than three combinations, the missing mutations were unsuccessful. ** Lowercase vv used to distinguish between V1016 and V410, with V410 being lower case. *** Eight individual mosquitoes did not produce a successful result and are not represented in this table.

## Data Availability

The raw data supporting the conclusions of this article will be made available by the authors on request.

## Supplementary Materials

The following supporting information can be downloaded at: https://www.mdpi.com/article/10.3390/insects16060551/s1, File S1: equations used to calculate *kdr* frequency and Wilcoxon Rank-Sum tests.
