# Supplementary material for: Detection of Di- and Tri-Locus kdr Mutations in Aedes aegypti and Aedes albopictus from Texas, USA, and the Implications for Insecticide Resistance
Source: Insects. 2025 May 23;16(6):551. doi: 10.3390/insects16060551 (PMC12192800; doi:10.3390/insects16060551)
Supplement: Supplementary file 1 [file insects-16-00551-s001.zip › insects-3532188-supplementary.pdf]

File S1

Mutation frequencies ($\hat{\rho}$) formula: $\hat{\rho} = \frac{2H_o+H_e}{n}$

where H_0 is the number of resistant homozygous alleles, H_e is the number of heterozygous alleles, and n is the number of samples.

The Wald interval: $(\hat{\rho} \pm z_{\alpha/2}\sqrt{\hat{\rho}(1-\hat{\rho})/n})$
